# Fast-speed and low-power-consumption optical phased array based on lithium niobate waveguides

**DOI:** 10.1515/nanoph-2024-0066

**Published:** 2024-03-28

**Authors:** Zhizhang Wang, Xueyun Li, Jitao Ji, Zhenxing Sun, Jiacheng Sun, Bin Fang, Jun Lu, Shaobo Li, Xiang Ma, Xiangfei Chen, Shining Zhu, Tao Li

**Affiliations:** National Laboratory of Solid State Microstructures, Key Laboratory of Intelligent Optical Sensing and Manipulation, Jiangsu Key Laboratory of Artificial Functional Materials, College of Engineering and Applied Sciences, 12581 Nanjing University, Nanjing, 210093, China; Optical Communication Research and Development Center, The 54th Research Institute of China Electronics Technology Group Corporation, Shijiazhuang, 050051, China

**Keywords:** thin-film lithium niobite, optical phased array, fast-speed, low-power consumption

## Abstract

Fast scanning speed and low-power consumption are becoming progressively more and more important in realizing high-performance chiplet optical phased arrays (OPAs). Here, we successfully demonstrated integrated OPAs with multiple waveguides channels based on thin-film lithium niobate-on-insulator (LNOI) platform. Specifically, two lithium niobate (LN) OPA chips have been implemented with 32 and 48 channels LN waveguides, respectively, enabled by electro-optic modulations, which showcases the low power consumption (1.11 nJ/π) and fast operation speed (14.4 ns), showing obvious advantage of the LNOI platform over others. As results, we experimentally achieved a beam steering with a 62.2° × 8.8° field of view (FOV) and a beam divergence of 2.4° × 1.2° for 32 channels, and a FOV of 40° × 8.8° and a beam divergence of 0.33° × 1.8° for 48 channels. This work also demonstrates the feasibility of LNOI platform in scalable OPA chips.

## 1 Introduction

Optical phased arrays (OPAs) have gained significant attention for applications in areas of light detection and ranging (LiDAR) [1], [2], [3], free-space communication [4], [5], imaging projection [6], [7], and remote sensing [8]. Compared to other optical beam forming and steering technologies [9], [10], [11], OPAs have been envisaged as a promising beam steering solution for an all-solid-state LiDAR due to its compact integration and scalability [12], [13], [14]. In particular, silicon (Si) platforms have made great success in integrated OPAs because of its mature fabrication and compatibility with complementary metal-oxide semiconductor (CMOS) processes [16], [17], [18], [19]. However, the two-photon absorption, high third-order nonlinearity, and large power-consumption in silicon limit the scalability and output beam power of OPAs based on Si platform [20], [21]. Silicon nitride (Si_3_N_4_) has been highlighted as an alternative platform to overcome those disadvantages by virtue of low nonlinearity and propagation loss [22], [23]; however, the scalability and power consumption of Si_3_N_4_ OPA have also been limited by the lower thermo-optical coefficient of Si_3_N_4_. Although the scheme of hybrid Si/Si_3_N_4_ integration has been proposed, it also suffers from considerable insertion losses as a function of applied phase shift [24], [25], [26], [27], [28].

Lithium niobate (LN) exhibits a wide transparent window (0.35–5 μm), low absorption loss, high second-order nonlinear optical coefficient, and relatively high Pockels electro-optic (EO) coefficient [29], [30], [31]. Especially, the electro-optic property of LN significantly enables the phase modulation efficiently, say, the high modulation speed and low losses at the same time. As early as 1974, OPAs based on the waveguides of epitaxial LN film have been reported by Tien et al. [32], achieving the beam deflection and the scan angle up to 4°. However, traditional bulk LN waveguides are fabricated mostly by metal diffusion, ion implantation, and proton exchange, resulting in large footprint of LN waveguides circuits. With the breakthroughs of the thin-film LN-on-insulator (LNOI) in fabrication techniques over the last few years, the LNOI has become a high-performance integrated photonics platform, which combines the superior optical properties and EO effect of LN material with large refractive index contrast of LNOI, making large-scale integrated OPAs possible in the future [33], [34], [35], [36], [37]. Recently, there have been successful LN OPAs reported [38], [39]; however, the major constraint arises from the very limited waveguide amount (only 16 channels). Moreover, the comprehensive performance of EO phase shifter and its advantages have not been revealed sufficiently.

Here, we demonstrate a monolithically integrated LN-OPA that reduces the power consumption while maintaining high operation speeds for integrated photonics chip. Employing the LNOI platform, the power consumption of an EO phase shifter is reduced to 1.11 nJ per π phase shift and response time is cut down to 14.4 ns. As a proof of concept, we demonstrated two LNOI OPA devices. One is a 32-channel array with a 62.2° × 8.8° field of view (FOV) in two dimensions by using EO modulation and wavelength sweeping, with a beam divergence of 2.4° × 1.2° at wavelength of 1550 nm, and the other is a 48-channel with sparse aperiodic arrays to decrease the lateral divergence of output beam down to 0.33°. Our work demonstrates LN-OPA as a scalable and power-efficient solution for various applications in the future, including all-solid-state LiDAR, free-space communication, etc.

## 2 Principle and design

**Figure 1:**
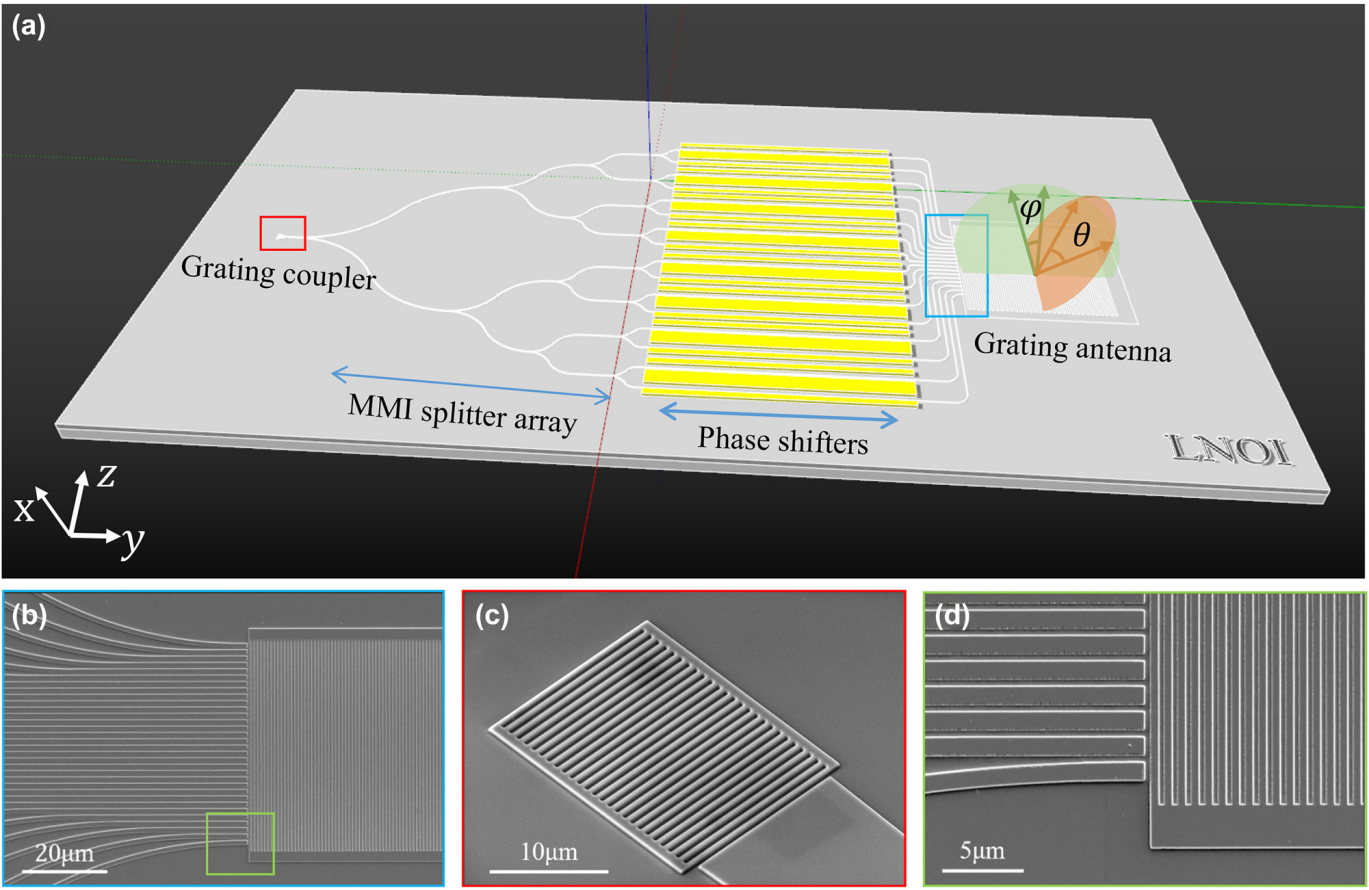
OPA chip based on LNOI. (a) Schematic illustration of the OPA for 2D beam steering. Scanning-electron microscope (SEM) images of the waveguide arrays and grating coupler are shown in (b), (c), and a close-up of the waveguide array in (d).

Figure 1(a) shows the schematic of the LN-OPA chip, which consists of a grating coupler, cascaded multi-mode interference (MMI) trees, EO phase shifters, and grating radiation antennas. Here, the OPA chip was fabricated in an X-cut LNOI platform (NANOLN) comprising a 600-nm-thick top LN film and buried oxide layer with a thickness of 2 μm. Ridge waveguides are designed 1 μm width with a 300 nm slab thickness and 300 nm ridge height (see Section S1 of the Supplementary Material). Firstly, the light around 1550 nm was controlled by polarization controller (PC) for maintaining TE polarization, coupled into the chip through the grating coupler (Figure 1(c)), and then split into 32 channels by a cascaded MMI splitter tree. Each channel passed through an EO phase shifter, which employs traveling-wave electrode design with length of 8 mm and the 4.5 μm pitch. At the end of the phase shifter array, the spacing of adjacent waveguides quickly decreases to 1.5 μm by bending the waveguide, as shown in Figure 1(b) and (d). The on-chip loss is a combination of about 0.55 dB/cm propagation loss, 0.06 dB loss per MMI splitter, and 8 dB for chip-fiber coupling loss at wavelength of 1550 nm (see Section S2 of the Supplementary Material).

Different from conventional waveguide grating antennas [2], [4], [40], a slab grating as a unitary device with an etched thickness same to waveguides [26] eliminates the need for a double etching process, simplifying the fabrication process. The multiple beam interferes in the slab grating and radiate out to space through the grating; therefore, the crosstalk among waveguides is negligible. The pitch of the waveguides can be reduced to 1.5 μm, theoretically achieving a scanning angle of 62°. In order to describe the emission angle of an OPA, beam steering angles have been defined in the lateral (*θ* axis) and longitudinal (*φ* axis) directions by EO phase modulation and operating wavelength sweeping, respectively. Depending on the linear phase difference Δ*ϕ* and the grating equation, the lateral and longitudinal sweeping angle *θ* and *φ* determined by [12]

(1) $$\sin\theta =\frac{\lambda_{0}\Delta \phi }{2\pi d},$$

(2) $$\sin\varphi =\frac{\Lambda n_{\text{eff}}-\lambda_{0}}{n_{0}\Lambda },$$

where Δ*ϕ* is the phase difference of adjacent waveguides, *d* is the spacing of waveguide array, *n*_*0*_ is the refractive index of the air, Λ is the slab rating period, *n*_eff_ is the effective index, and *λ*_0_ is the wavelength of the beam in the free space. When the spacing is larger than about half of a wavelength, the OPA with uniformly spaced emitters generates multiple grating lobes that can be expressed as $\pm \text{si}{\text{n}}^{-1}\left(\frac{\lambda }{2d}\right)$ . For the slab grating antenna, the antenna spacing is 1.5 μm, so theoretically a scanning range greater than ±31° can be achieved.

## 3 Results

**Figure 2:**
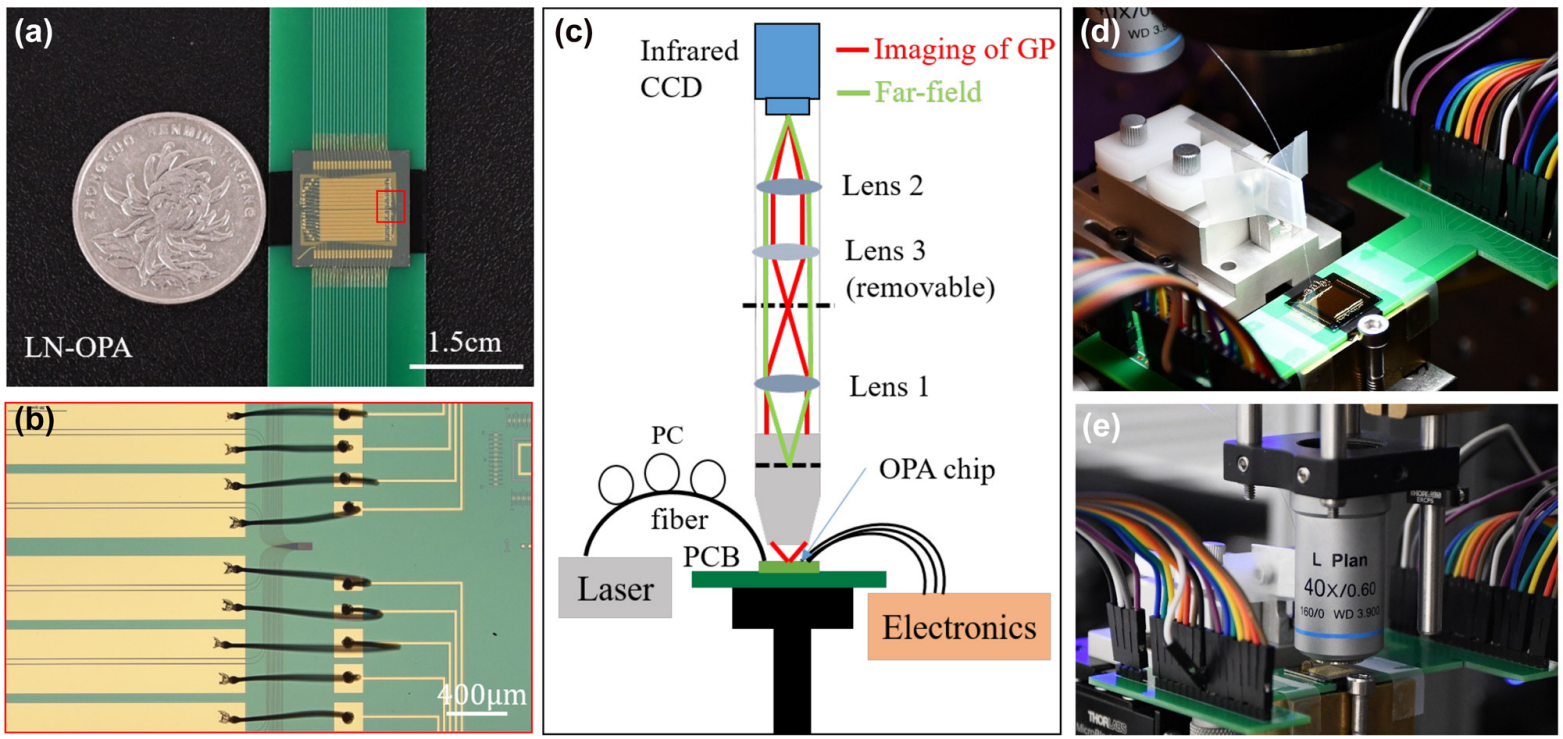
Images of the LN-OPA chip and the far-field characterization setup. (a) Photograph of the 1.5 cm × 1.5 cm LN-OPA chip wire bonded to a printed circuit board. (b) Microscopic photograph of wired-bonded electrodes and the region of grating antennas indicated by red box in (a). (c) Schematic of the imaging of grating plane (GP) and far-field measurement setup. (d) and (e) Photographs of fiber-to-grating coupling and testing in the experiment.

As shown in Figure 2(a), a 32-channel LN-OPA is fabricated on a 1.5 cm × 1.5 cm X-cut LNOI chip, which is wire-bonded on a printed circuit board (PCB) and controlled by digital-to-analog converters (DAC). Figure 2(b) shows the detailed microscopic photograph of wired-bonded electrodes and the region of grating antennas. Due to a total phased array aperture of 48 μm × 100 μm, we adopted an objective lens with a high NA (0.6) to achieve higher-performance far-field images and designed the emitting angle near 0° in the longitudinal direction at an input wavelength of 1550 nm. As shown in Figure 2(c), the imaging of grating plane and far-field measurement setup placed vertically was used to characterize the OPA beam steering, which brings convenience of switching between the grating plane and far-field images by employing/removing Lens 3. The grating plane imaging (all lenses; red rays) is used to align the imaging system to the region of emitter antennas, and then the Fourier image (Lens 1 and 2; green rays) corresponds to the far-field one. Figure 2(d) and (e) show photographs of the fiber-to-grating coupling and testing in the experiment, respectively. The infrared camera capturing the far-field image and DAC were connected to a computer simultaneously, and the output electric signals were changed by analyzing the far-field image. With the captured images as feedback, a particle swarm optimization (PSO) algorithm was employed to calibrate the initial phase and form a converged beam spot in the far field (see Section S3 of the Supplementary Material).

**Figure 3:**
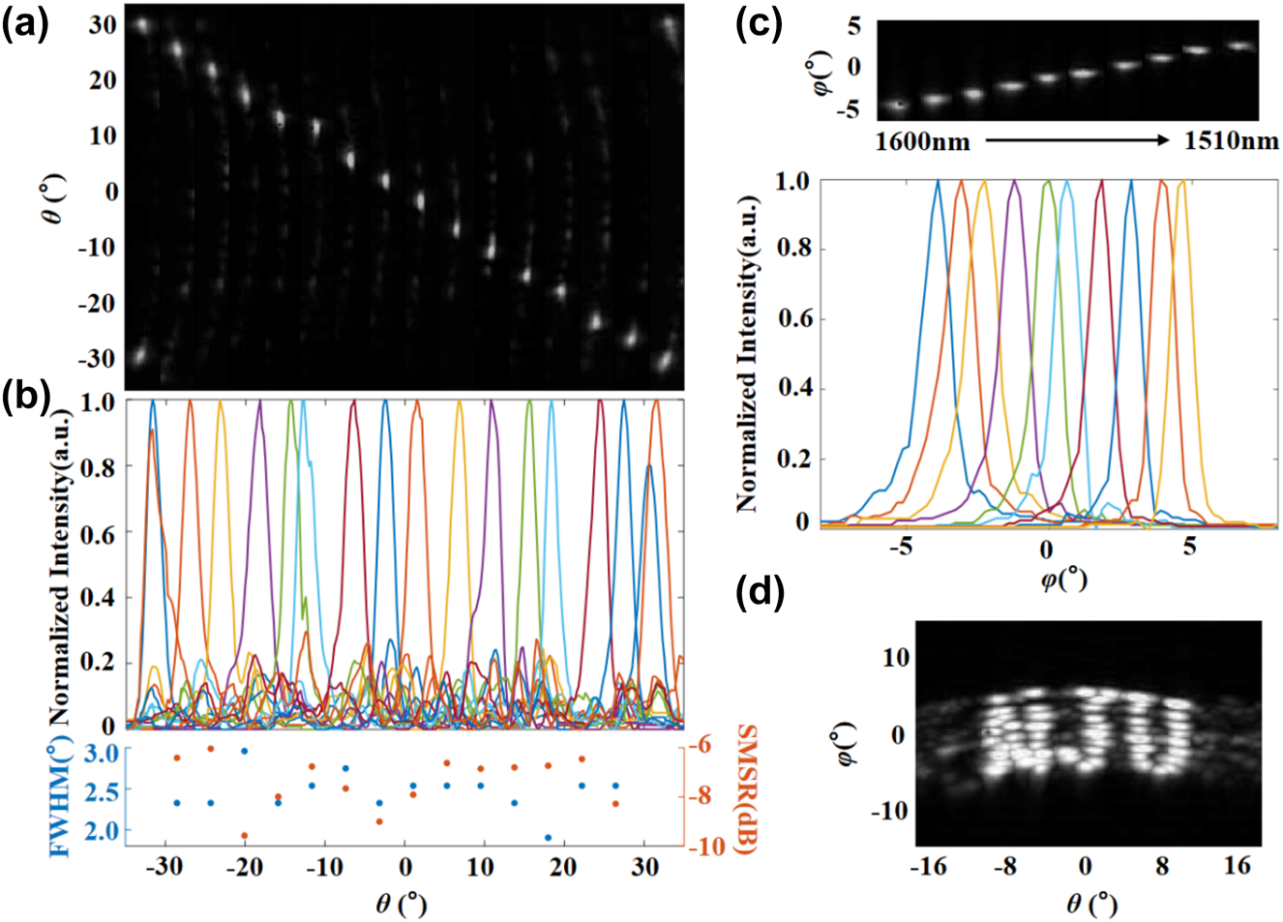
Characterization of far-field emissions of the 32-channel LN-OPA. (a) The spliced image of the far-field radiation pattern with the beam scanning range of ±31.1° in *θ* direction. (b) Measured normalized far-field intensity along the *θ* direction. Bottom inset: full width at half-maximum (FWHM) and side mode suppression ratio (SMSR) of each radiative beam with an average FWHM of 2.4° and the average SMSR of −7.4 dB. (c) Beam steering in *φ* direction and corresponding normalized intensity distribution by tuning the wavelength from 1510 nm to 1600 nm. (d) Normalized far-field beam steering with wavelength tuning and EO modulation, where an “NJU” pattern is formed covering a FOV of 23.2° × 8.8°.

As shown in Figure 3(a) and (b), we experimentally achieve aliasing-free beam steering from −31.1° to 31.1° at a fixed wavelength (1550 nm) in lateral direction, and the average side mode suppression ratio (SMSR) is <−7.4 dB when the beam is steered within ±31.1° range. As plotted in the bottom part of Figure 3(b), the steering beams are characterized to be with an average full width at half-maximum (FWHM) of 2.4°, which is mainly limited by the size of the radiation aperture (48 μm) in lateral direction. By sweeping the laser wavelength from 1510 nm to 1600 nm, the emission light can achieve a FOV of 8.8° and an FWHM of 1.2° in longitudinal direction (see Figure 3(c)). The divergence in the longitudinal direction can be further improved by employing a well-controlled shallow grating etch. So far, the capability of lateral and horizontal scanning of OPA has been individually demonstrated with a FOV of 62.2° × 8.8° and an FWHM beam size of 2.4° × 1.2°. In addition, we utilize the wavelength tuning to slice the 2D pattern and perform lateral scanning through EO modulation to condense three letters of “NJU” with a FOV of 23.2° × 8.8° as shown in Figure 3(d), which verifies the capability of the LN-OPA for two-dimensional beam scanning. It is a nonignorable phenomenon that the far-field beam spot has a process of unstable state when the modulation voltage applied, which would account for the photorefractive (PR) effect in LN material. We also find that the far field beam spot can be a stabilized and repeatable state when we gradually increase the scan rate (the influence of PR effect can be reduced by removing the dielectric cladding [41]). In fact, our results have confirmed that the PR effect is effectively mitigated when the scan rate is higher than the response time of PR effect (see below and Section S5 of the Supplementary Material).

**Figure 4:**
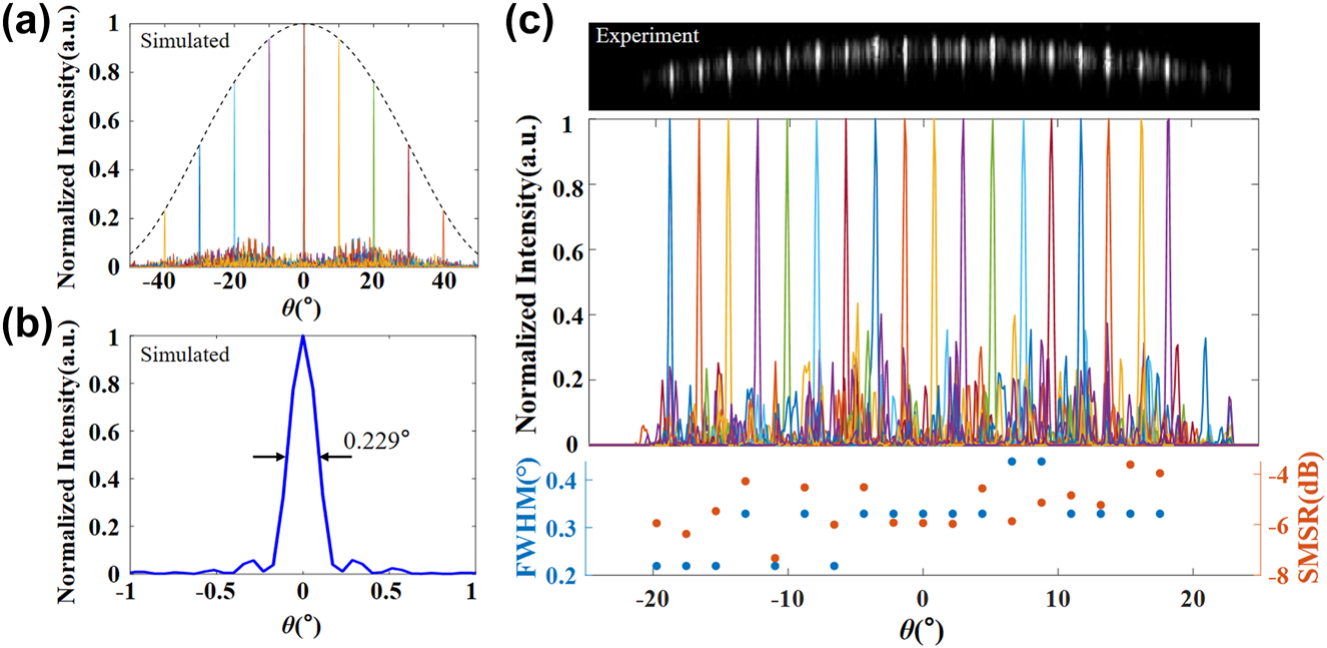
Characterization of far-field emissions of the 48-channel LN-OPA. (a) Simulated far-field intensity distribution at different steering angles of the proposed aperiodic antennas. (b) Magnified view of the simulated far-field beam at 0° with an FWHM beam size of 0.229°. (c) Characterization of far-field emissions of aperiodic spacing antennas of 48-channel LN-OPA. Top: the sliced profile of far-field pattern. Middle: corresponding normalized intensity distribution as a function of *θ*. Bottom: FWHM and SMSR of each radiative beam, showing an FWHM beam size of 0.33° and average SMSR of about 5 dB.

In order to achieve smaller beam divergence in *θ*, we extend the phase channels from 32 to 48 and adopt a sparse aperiodic arrangement of arrays to achieve a larger radiation aperture, which suppresses the higher order diffractive beams at the expense of the reduced optical power in the main beam compared to the background (see Section S4 of the Supplementary Material). As demonstrated above, the divergence angle in *θ* is limited by the number of phase channels and total footprint of emission aperture. As a result, a genetic algorithm (GA) is applied for a higher SMSR by optimizing the position of each channel and the sparse aperiodic arrangement 48-channel LN-OPA with an average pitch of 5.6 μm and the total aperture size of 410 μm is fabricated and experimentally investigated, with a simulated result of the FOV of ±30° with the SMSR of 11 dB–8 dB and the FWHM of 0.229° for beam steering of 0° (see Figure 4(a) and (b)). The simulated far-field intensity distribution at different steering angles is presented in Figure 4(a), and in the experiment, the measured results are normalized. As shown in Figure 4(c), the scanning FOV of the LN-OPA achieves ±20° and the FWHM beam size is effectively narrowed down to about 0.33° with SMSR of about 5 dB. The optimization for divergence has a decrease in FOV and SMSR, which is mainly due to the sparse aperiodic design of the waveguides. Nevertheless, it is quite promising to improve the performance of FOV and SMSR by further increasing the number of channels. As results, the FWHM is decreased to one-sixth compared with the uniform spacing antennas of 32-channel OPA thanks to the sparse aperiodic waveguide arrays, providing a feasible solution to decrease the lateral divergence and improve the performance of the OPA.

**Figure 5:**
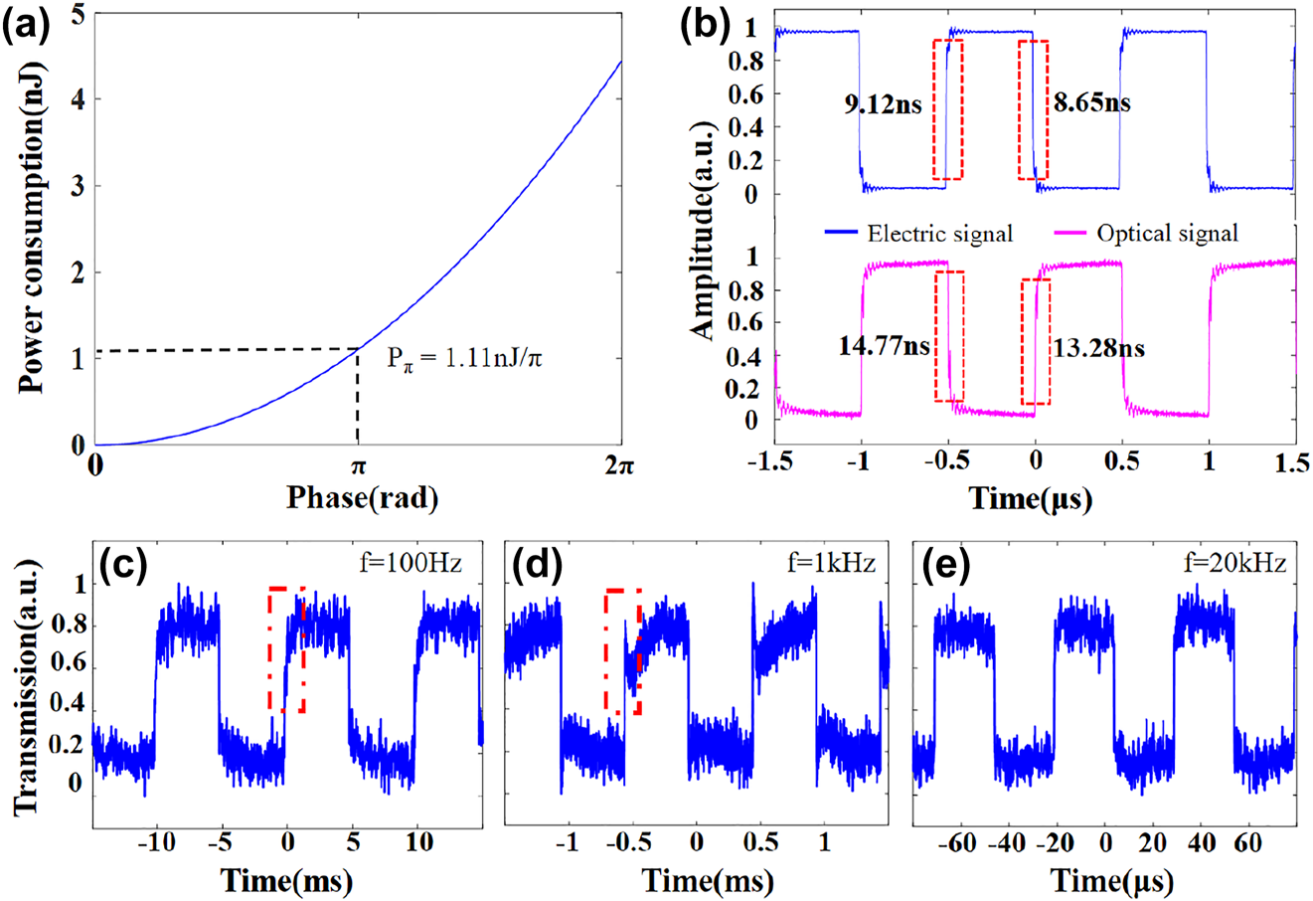
Measured power consumption and response time performance of the phase shifter. (a) The power consumption of a phase shifter as a function of phase difference between two arms of Mach–Zehnder interferometer. (b) Measured response time when applying a square voltage signal, indicating a switching speed of about 14 ns. (c–e) Optical response when rectangle waveform signals of 100 Hz, 1 kHz, and 20 kHz are applied on the phase shifter.

Compared with conventional OPAs based on other material platforms, the LN-OPA possesses significant advantages of power consumption and operation speed owing to the excellent EO effect of LN material. For the operation speed of the phase shifter, the rising and falling time of LNOI Mach–Zehnder interferometer (MZI) phase shifter have been tested the power consumption and response time of the OPA. Compared with conventional OPAs based on other material platforms, the LN-OPA possesses significant advantages of power consumption and operation speed owing to the excellent EO effect of LN material. For the operation speed of the phase shifter, the rising and falling time of LNOI MZI phase shifter have been tested to reflect the power consumption and response time of the OPA. The MZI phase shifter is considered to be equivalent to a capacitor and its capacitance is measured to be 39.5 pF by an inductance–capacitance–resistance (LCR) digital bridge tester. With the measured half-wave voltage (π phase shift at 7.5 V by each phase shifter) *V*_π_ of 7.5 V, the power consumption as a function of the induced phase shifts between two arms of MZI is plotted in Figure 5(a) and calculated to be 1.11 nJ/π per channel. It is worth noting that the energy stored in the MZI is almost consumed by peripheral circuits, and thus the actual power consumption of the LN-OPA should be much lower than that calculated above (see Sections S5 & S6 of the Supplementary Material). A 1 MHz square voltage signal is applied on the phase shifter, and the modulated light is transformed into electric signals by a photodetector (PD) connected with an oscilloscope (see Figure S7 of the Supplementary Material). Figure 5(b) illustrates the waveforms of input electrical signal and response optical signal and indicates the rising and falling time up to 13.28 ns and 14.77 ns, which exhibits extremely fast optical response time. In the experiment, a drift of optical response caused by the PR effect is also observed. When a series of square waveform signals of various frequency are applied on the LN MZI phase shifter, the optical outputs present a slow rise of several milliseconds and a rapid peak within about 50 μs (see red dashed box in Figure 5(c) and (d)). When the frequency comes to 20 kHz, the drift is mitigated, indicating the proper operating time for OPA to be free from the impact of the PR effect (see Figure 5(e)).

## 4 Discussion and conclusion

Table 1 shows a comparison of performance metrics of the OPAs based on different platforms. Conventional OPAs have been predominantly restricted by operation speed (around μs) and excessive power consumption to modulate the phase. In this work, by leveraging the exceptional EO properties of LN, the proposed LN-OPA can operate at ultra-high speed, almost three orders of magnitude over other OPAs. Besides, the power consumption of the LN-OPA is comparable to that of the OPAs based on plasma dispersion effect (which however suffers from high optical loss) and far outperforms the OPAs based on thermo-optics effect. The LNOI platform provides a compact, low-power-consumption and scalable to implement high-performance integrated OPA chips. However, due to the limitation of the number of channels, the FOV and the FWHM of LN OPAs are still inferior compared to other platforms, as reported in Ref. [38], [39]. In this work, we expanded the number of channels to 32 and compressed the spacing between waveguide arrays to sub-wavelength dimensions, achieving a larger FOV of 62°. Furthermore, by further expanding the number of channels to 48 and employing a sparse aperiodic arrangement of arrays, we reduced the divergence angle to 0.33° with a little sacrifice in FOV. Although the scale and divergence of LN-OPA just have conventional performances, it should be mentioned that the LN-OPA is chosen to be with 32 and 48 channels, respectively, for a proof of concept in this work. In the future, we will further increase the number of phase channels and adopt shallow etching to improve the aperture size as well as the performance of FOV and SMSR. More importantly, owing to the incomparable advantages in operation speed and power consumption, the LN-OPA is of potential scalability and higher performances.

**Table 1:** The comparison of performance metrics of OPAs based on different platforms.

Platform	Principle	Power consumption	Speed	FOV (*a*° × *b*°) (SMSR)	FWHM (*a*° × *b*°)	Channels	Ref.
SOI	Plasma dispersion effect	2 μW/π	30 μs^a^	±50 × 17 (10 dB)	0.01 × 0.039	8192	[25]
SOI	Thermo-optics effect	22 mW/π	–	±33 × 3.3 (7 dB)	–	24	[15]
Si_3_N_4_–Si	Thermo-optics effect	20 mW/π	22.8 μs	±48 × 14	2.3 × 2.8	32	[26]
Si_3_N_4_–Si	Plasma dispersion effect	1.8 μW/π	30 μs	±70 × 19.4 (7.4 dB)	0.021 × 0.1	128	[27]
Si_3_N_4_–Si	Thermo-optics effect	–	–	±70 × 16.7 (11 dB)	0.051 × 0.016	256	[28]
SOI	Thermo-optics effect	7 mW/π	–	±70 × 13.5 (13.2 dB)	2.1 × 0.08	64	[16]
LNOI	Pockels EO effect	13.5 pJ/π^b,c^	0.23 ns^c^	±12 × 8 (10 dB)	2 × 0.6	16	[38]
LNOI	Pockels EO effect	0.33 nJ/π^b^	0.4 ns^d^	±25 × 8.6 (5.1 dB)	0.73 × 2.8	16	[39]
LNOI	Pockels EO effect	1.11 nJ/π^b^	14.4 ns	±31.1 × 8.8 (7.4 dB)	2.4 × 1.2	32	This work
		±20 × 8.8 (5 dB)	0.33 × 1.8	48			

^a^The results is for a packaged OPA in a LiDAR configuration (using DAC to drive the phase shifters). ^b^For Pockels EO effect, the power consumption is description by energy change. ^c^The simulated results (the simulated modulator capacitance). ^d^For a traveling-wave electrode modulator, pads are connected to the load resistors (50 Ω).

In summary, we have demonstrated monolithically integrated optical phased arrays based on LNOI platform, which decreases the power consumption to the level of nanojoule per π phase shift and improves the operation speed to nanosecond simultaneously. As a proof of concept, two LN-OPA chips have achieved beam steering of a 62.2° × 8.8° FOV with the beam divergence of 2.4° × 1.2° for 32 channels and 40° × 8.8° FOV with the beam divergence of 0.33° × 1.8° for 48 channels, respectively. By using LNOI platform, the scalability of OPAs limited by the high power consumption of their embedded large number of phase shifter would be remarkably improved. With the advantage of scalability and low power consumption, the LNOI photonics platform facilitates the prospect of a power-efficient, compact, and large-scale integrated OPA for wide applications including long-range LiDAR, autonomous vehicles, and free-space communications.

## Supplementary Material

Supplementary Material Details
