# Supplementary material for: Fast-speed and low-power-consumption optical phased array based on lithium niobate waveguides
Source: Nanophotonics. 2024 Mar 28;13(13):2429–36. doi: 10.1515/nanoph-2024-0066 (PMC11501545; doi:10.1515/nanoph-2024-0066)
Supplement: Supplementary file 1 — Supplementary Material Details [file j_nanoph-2024-0066_suppl_001.docx]

**Supplementary Material**

**Fast-speed and low-power-consumption optical phased array based on lithium niobate waveguides**

Zhizhang Wang, ^a,†^ Xueyun Li, ^a,†^ Jitao Ji ^a^, Zhenxing Sun ^a^, Jiacheng Sun ^a^, Bin Fang ^a^, Jun Lu ^a^, Shaobo Li,^b^ Xiang Ma,^b^ Xiangfei Chen ^a^, Shining Zhu ^a^, and Tao Li ^a,*^

a Nanjing University, National Laboratory of Solid State Microstructures, Key Laboratory of Intelligent Optical Sensing and Integration, Jiangsu Key Laboratory of Artificial Functional Materials, College of Engineering and Applied Sciences, Nanjing, China, 210093

b Optical Communication Research and Development Center, the 54th Research Institute of China Electronics Technology Group Corporation, Shijiazhuang, China, 050051

# Section 1: Fabrication process of LN-OPA

A commercially available X-cut lithium niobate-on-insulator (LNOI) wafer (NANOLN) with a 600nm thick lithium niobate (LN) layer and a 2 μm buried silicon dioxide is first cleaned and a layer of ma-N2405 is spin-coated on the LN thin film as a mask. The waveguide patterns are defined by an E-beam lithography (EBL) process on the resist and transferred 300 nm deep into LN with an optimized argon plasma and CHF_3_ in an inductively coupled plasma (ICP) etching system subsequently. After removing the resist and cleaning the LN sidewall, two layers of LOR5B film and AZ5214 film are spin-coated and followed by an ultraviolet (UV) exposure to define the electrode patterns. After development, a 400 nm thick gold film is deposited onto the wafer and then a lifted-off process transfers the patterns. Finally, the fabricated chip is mounted onto a printed circuit board (PCB) with two-step wire-bonding which connects the electrodes of phase shifters to the surrounding pads and the pads to the PCB, respectively (Fig. S1).


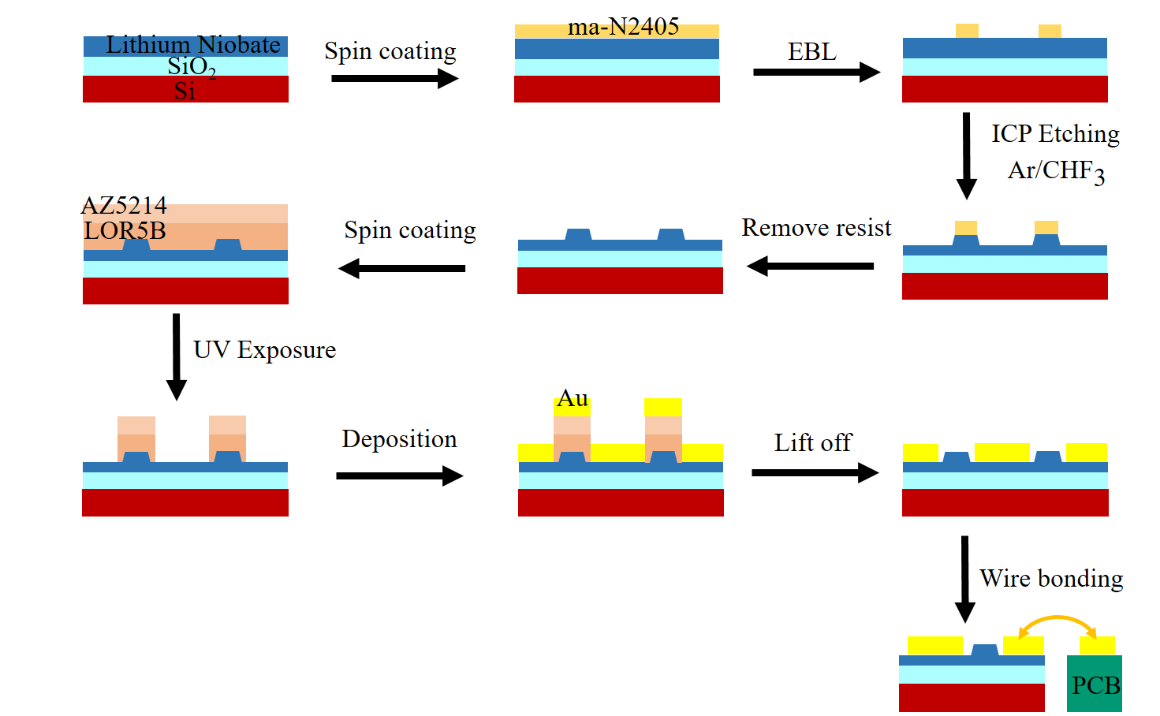


**Fig. S1** The fabrication procedure of the proposed LN-OPA.

# Section 2: Loss measurement


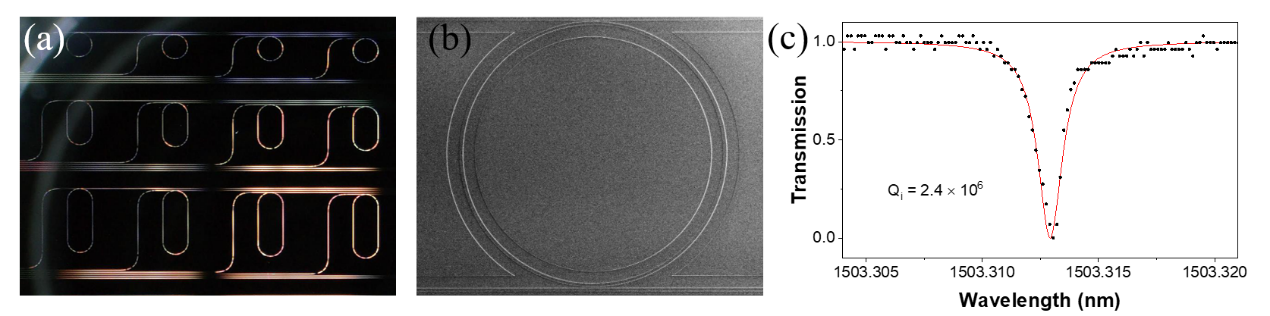


**Fig. S2** (a) The optical images of the fabricated LN micro-ring and race-track resonators. (b) The scanning electron images of the LN micro-ring resonator and (c) the corresponding transmission.

To characterize the propagation loss of LN waveguide, a series of LN micro-ring and race-track resonators with different lengths were designed and fabricated. Figure S2(a) and S2(b) display the optical microscope images of the fabricated samples and one of the micro-ring resonators, respectively. The Q-factor of the LN micro-ring resonator was measured to be 2.4×10^6^, as depict in Fig. S2(c). With Q-factor, the propagation loss of LN waveguides could be calculated according to the following equation

$$Q=2\pi n_{g}/(\lambda\alpha)$$

where *n_g_* is the group index of refraction and α denotes the propagation loss. Therefore, the average propagation loss of the fabricated LN waveguides (with height of 600 nm and etching depth of 300nm) is estimated to be 0.55dB/cm.


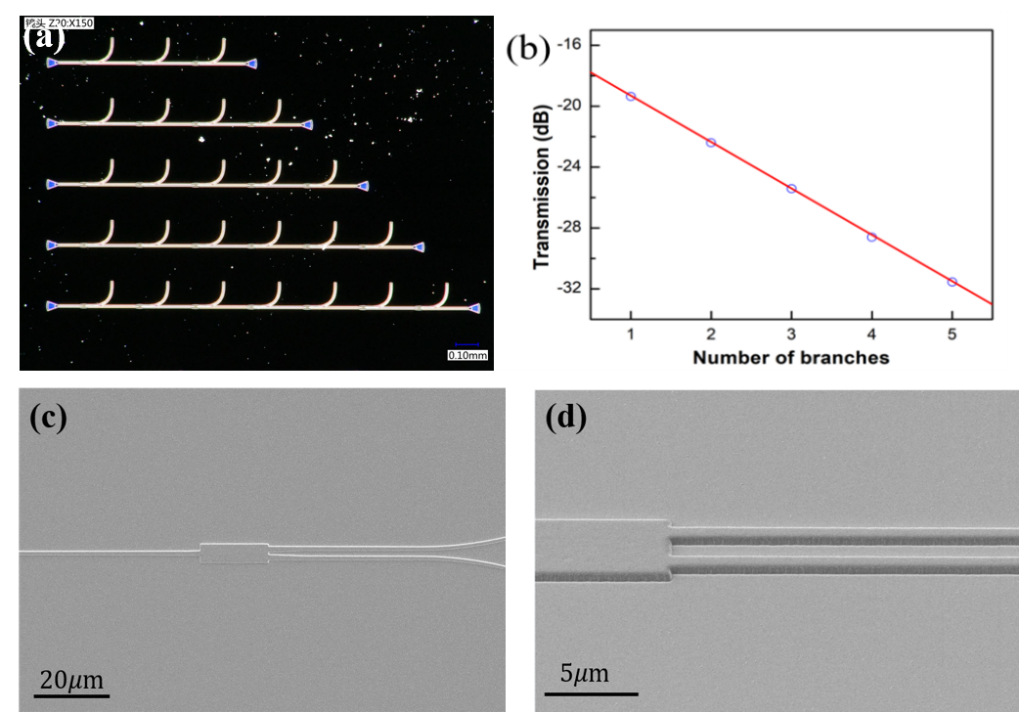


**Fig. S3** (a) The optical microscope image of the cascaded MMI couplers. (b) Transmission of the cascaded MMI couplers as a function of the number of branches (blue dots) and the linear fitting line (red solid line). (c) The scanning electron images of the fabricated MMI coupler. (d) The enlarged view of the MMI coupler.

In addition to propagation loss, the excess loss of multi-mode interference (MMI) couplers has also been investigated through establishing a cascaded configuration. The single MMI coupler and the cascaded MMI couplers were fabricated as shown in Fig. S3(a) and S3(c-d). Figure S3(b) plots the transmission as a function of the number of branches at the wavelength of 1550 nm. Through linear fitting, the slope of the fitting line can be extracted to be -3.06dB, indicating the excess loss of the MMI coupler to be 0.06dB.

# Section 3: Far field radiation calibration


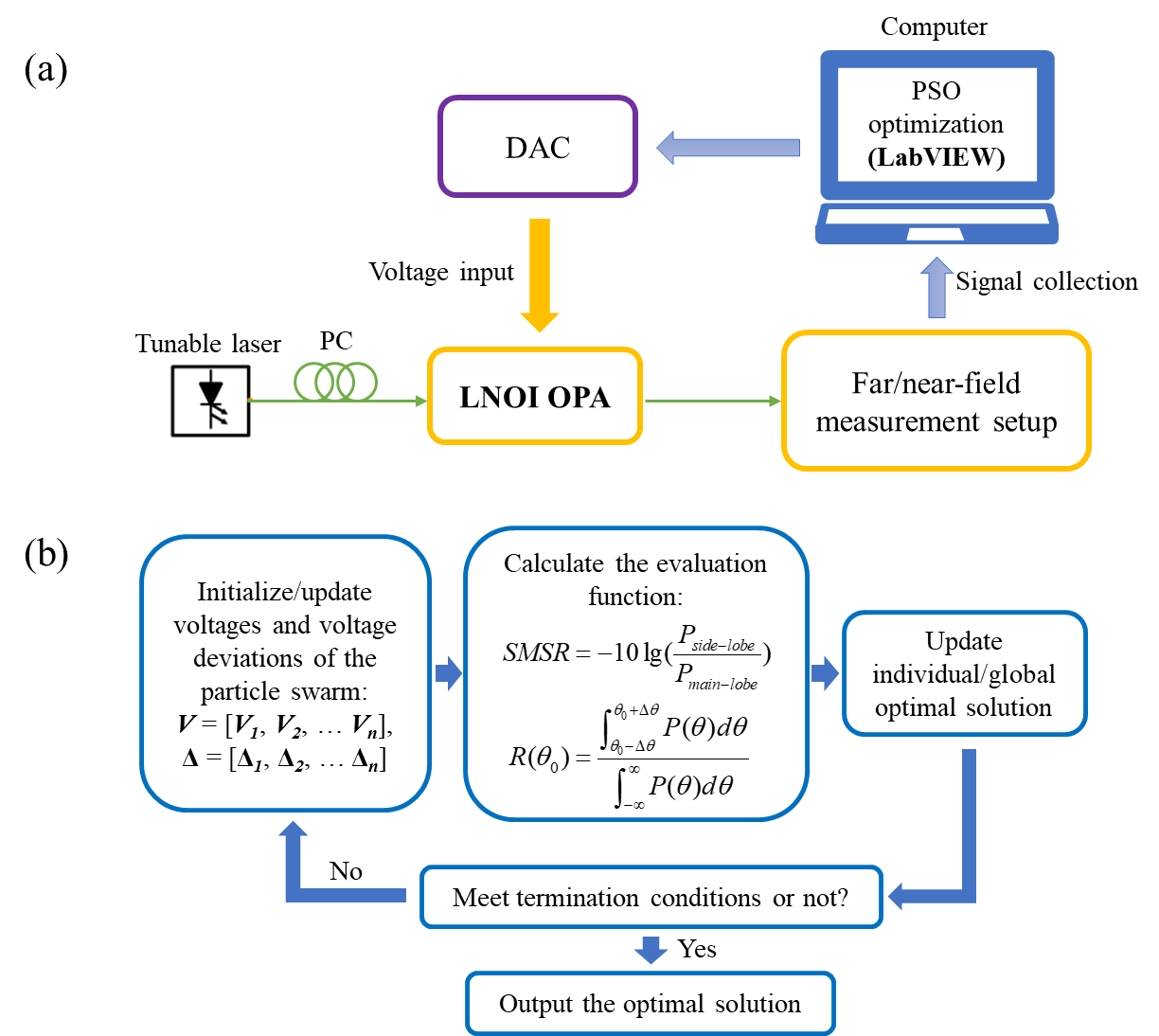


**Fig. S4** (a) The testing system for phase calibration, utilizing a particle swarm optimization (PSO) algorithm implemented in NI LabVIEW code. (b) The flowchart of PSO algorithm. DAC: digital – analog converter. PC: polarization controller.

In the experiment, due to the phase distortion caused by fabrication imperfection such as rough sidewall of LN waveguides and processing deviation of bending waveguides, phase calibration at the emitter is necessary to suppress side lobes and improve steering efficiency. Figure S4(a) illustrates the testing system for the phase calibration based on particle swarm optimization (PSO) algorithm implemented in NI LabVIEW code. The optimization is executed on a computer connected with the near infrared charge coupled device (CCD, RAPTOR/OW1.7-CL-640) and the digital-to-analog converter (DAC, NI9264), acquiring far-field images and updating electric voltages to the LN-OPA respectively.

The PSO algorithm starts with a set of arbitrary locations (corresponding to voltages applied to the phase shifters) and a set of arbitrary velocities (corresponding to voltage deviations) which compose the property of particle swarm (see Fig. S4(b)). The far-field images after Fourier transformation by far/near-field measurement setup are taken and evaluated for their side mode suppression ratio (SMSR) and central energy ratio$R(\theta_{0})$ as the composite evaluation function which gives the criterion to update the individual (global) optimal solution in the optimization process. The updated velocity set and the location set in the next iteration are calculated by

$\Delta_{i+1}=w\times\Delta_{i}+c_{self}\times r_{1}\times(p_{best}-V_{i})+c_{social}\times r_{2}\times(g_{best}-V_{i})$, (S1)

$V_{i+1}=V_{i}+\Delta_{i+1}$, (S2)

where *V_i_* and $\Delta$*_i_* is the location set and the velocity set in the *i*-th iteration, *w* is the predefined inertia weight, *c_self, social_* is the predefined individual/social factor, *r_1,2_* is the random number between 0 and 1, *p_best_* and *g_best_* are the individual and global optimal solution respectively. When the termination conditions are satisfied, the optimization program will be stopped, and corrected far-field beam spot at the expected steering angle is acquired when the optimal voltage array applied.


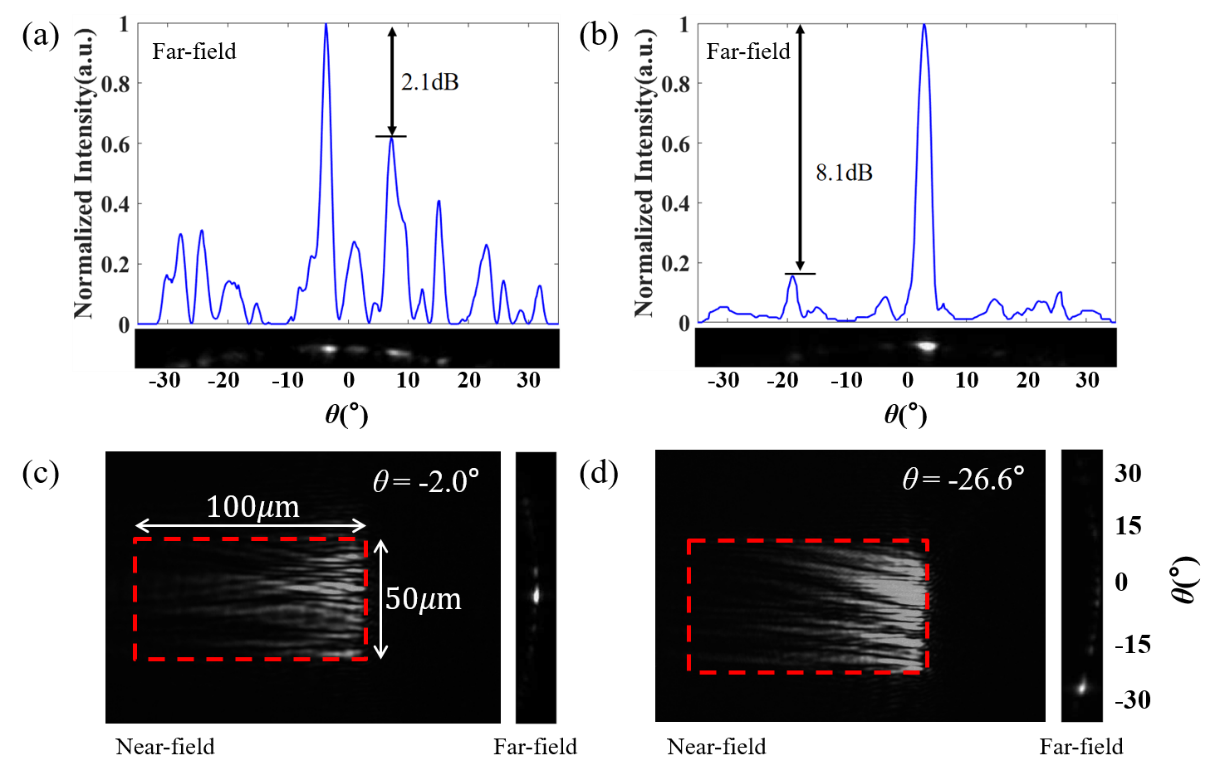


**Fig. S5** (a), (b) The far-field intensity distribution before and after calibration respectively. (c), (d) Near/far-field images with steering angle of -2.0°and -26.6°after phase calibration. Red dashed line indicates the area of grating antennas of 48 μm × 100 μm size.

Due to the near/far-field measurement system is established, the phase of LN-OPA is calibrated for desired steering angle and validated its performance. Figure S5(a) and S5(b) present the comparison of far-field intensity before and after calibration, showing an improvement from 2.1 dB to 8.1 dB. After calibration process, the near-field and far-filed images are respectively captured. As shown in Fig. S5(c) and S3(d), optical field distribution in the near-field has tilted wavefront at the different angles of 2.0°and -26.6°, which corresponds to the measured beam deflection angles in the far field.

# Section 4: The optimization of aperiodic spacing antennas


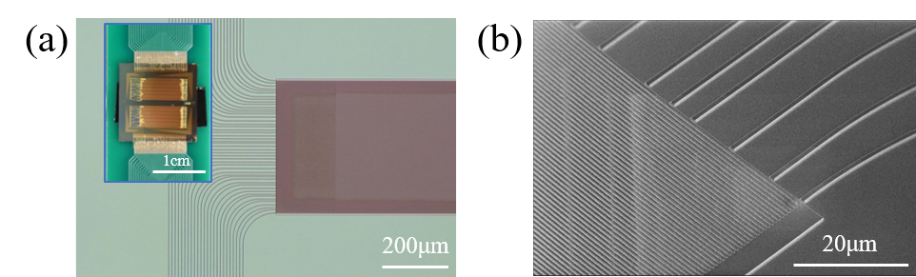


**Fig. S6** (a) Microscopy image of the emitting grating of the fabricated LN-OPA chip based on the proposed aperiodic spacing antennas design. Inset: holistic photograph of the LN-OPA chip. (b) Scanning-electron microscope (SEM) image of part of the antennas.

In the application, the beam divergence is a vital metric associated with detection range and precision and expected to be as small as possible. The direct approach to decrease the divergence angle is enlarging the radiative aperture size, such as extending channels and increasing the pitch between adjacent waveguides. Limited by footprint of the chip and the fabrication complexity, number of channels cannot dramatically rise up, and therefore the sparse aperiodic arrangement in waveguides design is the feasible way for OPAs. We utilize a sparse aperiodic spacing design to suppress grating lobes and a genetic algorithm (GA) is applied for a higher SMSR by optimizing the position of each channel with an average pitch in the range of 3λ to 8λ. The optimized 48-channel waveguide array has a size of 410 μm in the direction of phase modulation and an average pitch of 5.6λ. The detailed positions of every waveguide in the aperiodic antennas are listed in Table S1. Simulation results show that the aperiodic antennas is capable to steer beam within ±30° FOV maintaining a SMSR more than 8 dB and the FWHM at the 0° emitting is 0.229° (see Fig. 6(a) and 6(b)). As the proof of concept, we fabricated the LN-OPA with the sparse aperiodic spacing antennas design and experimentally tested its performance. Figure S6(a) and S6(b) show the microscopy image of the emitting grating and the SEM photograph of the details at the antennas respectively with the full-view of the fabricated LN-OPA chip inset in Fig. S6(a).

**Table S1** List of the positions of waveguide in the 48-channel aperiodic antennas

| Number | Position  (μm) | Number | Position  (μm) | Number | Position  (μm) | Number | Position  (μm) |
| --- | --- | --- | --- | --- | --- | --- | --- |
| 1 | -205.1 | 13 | -108.3 | 25 | -1.8 | 37 | 111.3 |
| 2 | -198.0 | 14 | -102.0 | 26 | 9.6 | 38 | 117.7 |
| 3 | -188.3 | 15 | -92.3 | 27 | 18.9 | 39 | 128.7 |
| 4 | -178.8 | 16 | -80.0 | 28 | 27.8 | 40 | 138.1 |
| 5 | -173.7 | 17 | -69.6 | 29 | 37.1 | 41 | 147.0 |
| 6 | -167.3 | 18 | -62.9 | 30 | 47.7 | 42 | 151.9 |
| 7 | -155.2 | 19 | -51.6 | 31 | 55.3 | 43 | 162.5 |
| 8 | -147.8 | 20 | -44.1 | 32 | 67.6 | 44 | 168.3 |
| 9 | -139.0 | 21 | -33.8 | 33 | 79.9 | 45 | 180.2 |
| 10 | -131.7 | 22 | -24.6 | 34 | 87.5 | 46 | 189.1 |
| 11 | -121.7 | 23 | -16.5 | 35 | 92.3 | 47 | 195.0 |
| 12 | -114.4 | 24 | -11.5 | 36 | 100.1 | 48 | 205.1 |

# Section 5: The measurement of response time


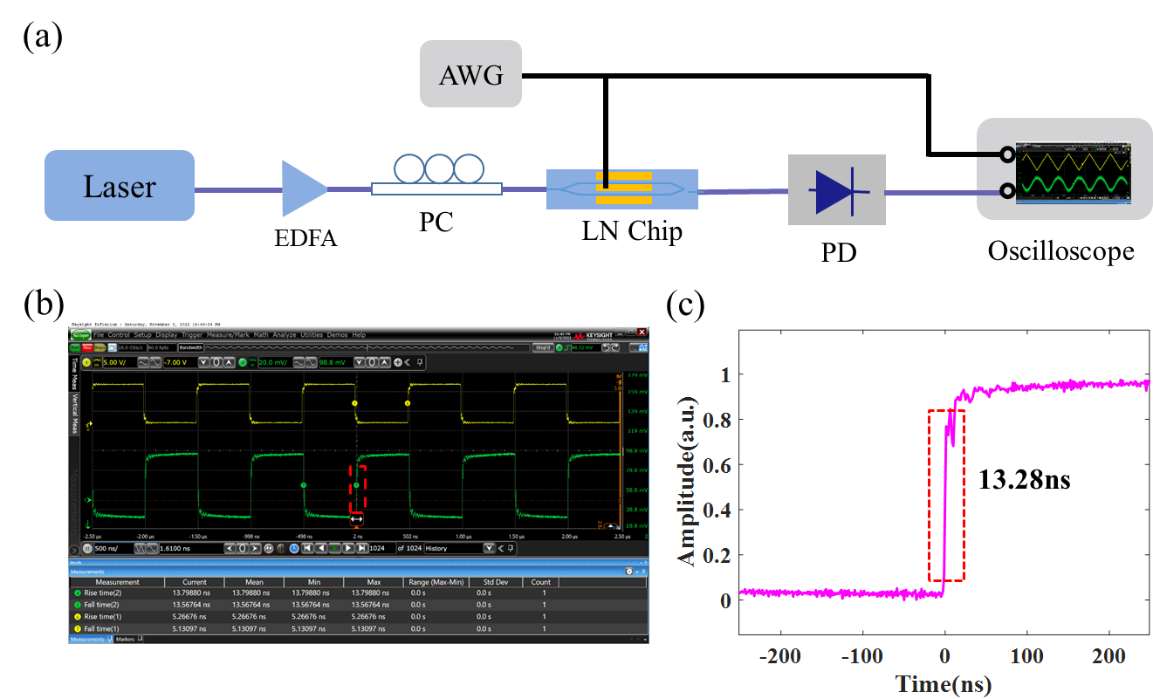


**Fig. S7** (a) Experimental setup for response time and half-wave voltage measurement. EDFA: erbium-doped fiber amplifier. PC: polarization controller. AWG: arbitrary waveform generator. PD: photodetector. (b) Oscilloscope screenshot of the response time. (c) Magnified view of a rising edge, indicating the switching time of 13.28 ns.

Regarding the speed of the OPA, we tested the rising and falling edges of the Mach-Zehnder interferometer (MZI) phase shifter. The experimental setup for response time measurement is presented in Fig. S7(a). Light is emitted from a tunable laser (Keysight 8164B), amplified by an erbium-doped fiber amplifier (EDFA) and then passes through a polarization controller (PC) before being injected into the chip. An arbitrary waveform generator (AWG, Tektronix AFG3251) controls the electric voltage on the electrodes of the MZI phase shifter and modulates the light passing through the chip which is detected by a photodetector (PD) and an oscilloscope (Keysight MXR054A). With a 1 MHz square waveform signal of half-wave peak to peak voltage applied on the chip, the average rising time of 13.28 ns and falling time of 14.77 ns are achieved as presented in Fig. 7(a) and Fig. S7(b) and the magnified view of a rising edge is exhibited in Fig. S7(c).


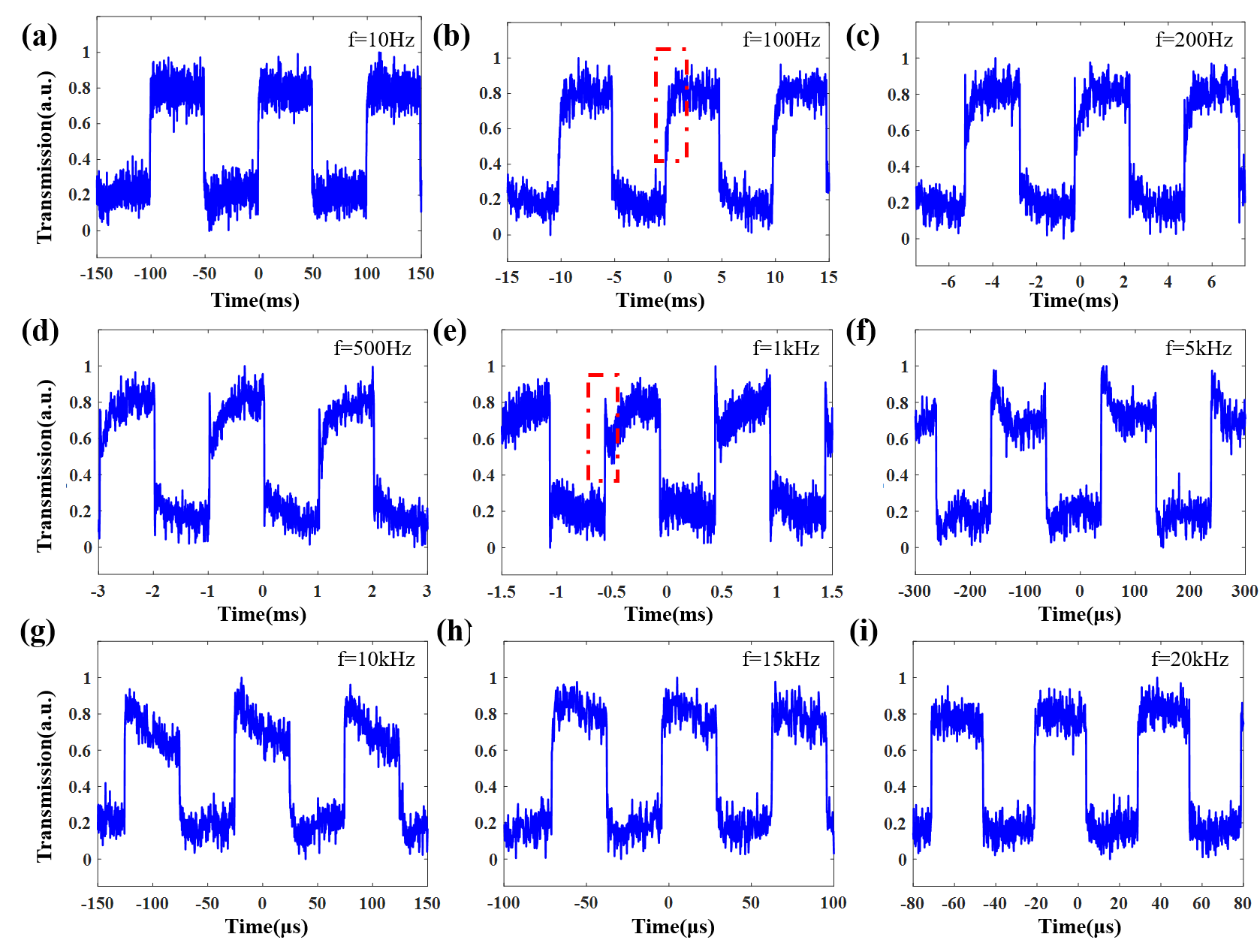


**Fig. S8** Voltage drifts caused by photorefractive effect. Rectangle waveform signals of different frequency from 10 Hz to 20 kHz and identical peak-to-peak voltage is applied on the phase shifter, corresponding optical response shown in (a)-(i) respectively.

As an inherent property of LN, the photorefractive (PR) effect impedes its usage in practical application and appears in our experiment of OPA testing. The PR effect induces the drift of far-field beam spot, which is mitigated by increasing the scanning rate. To further investigate the relation between the drift and the scanning rate for drift-free operation, we applied a series of square waveform signals of identical peak-to-peak voltage and various frequency from 10 Hz to 20 kHz on the LN MZI phase shifter and detected the optical outputs. Results show that when the voltage changes, the power of transmitted light presents a slow rise of several milliseconds (see red dashed box in Fig. S8(b)) after a rapid peak within about 50 μs (see red dashed box in Fig. S8(e)). With the frequency increasing, the drift is gradually mitigated and vanished at the frequency of 20 kHz, where the impact of PR effect can be effectively eliminated.

# Section 6: The principle and measurement of power consumption


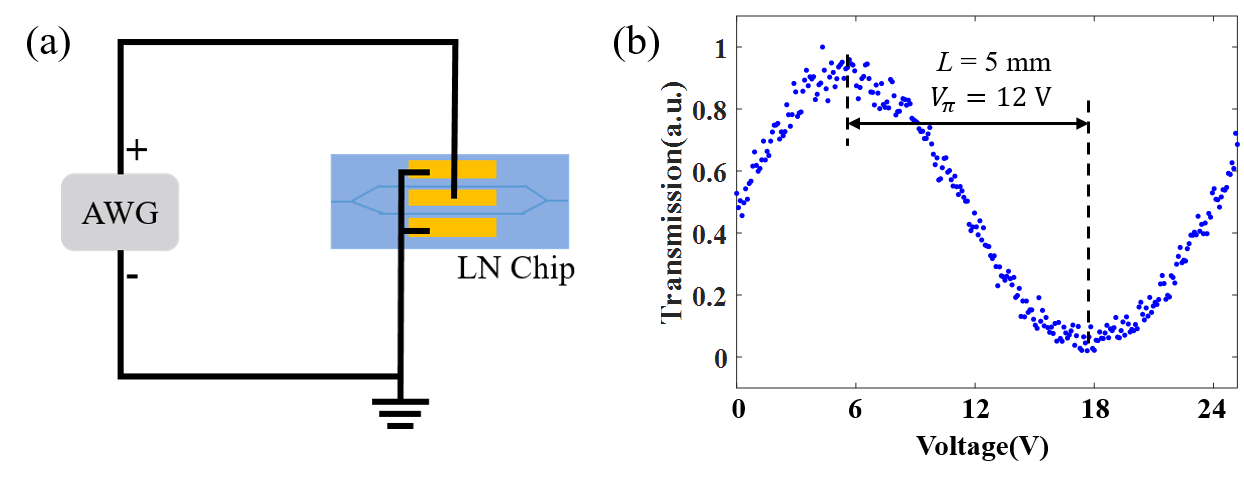


**Fig. S9** (a) The equivalent circuit of the phase shifter which can be seen as a capacitor. (b) Measured normalized optical transmission of a 5 mm phase shifter, indicating the *V_π_* value of 12 V.

For traditional OPAs based on thermo-optics effect, the power consumption is predominantly from the heat dissipation. By contrast, the phase shifter of LN-OPA is based on Pockels electro-optics effect and equivalent to a capacitor (the equivalent circuit is shown in Fig. S9(a)), which have inherently no energy dissipation but energy change. The energy changed in the phase shifter can be calculated by

$E=\frac{1}{2}CV_{rms}^{2}$, (S3)

where *C* is the capacitance and *V_rms_* is the root-mean-square voltage. With the measurement setup illustrated in Fig. S7(a), we experimentally acquired the half wave voltage of 12 V for a *L*=5 mm phase shifter, implying the voltage-length product of (shown in Fig. S9(b)), and correspondingly the half wave voltage of the *L*=8 mm LN MZI phase shifter which has the same configuration as LN-OPA phase shifters is 7.5 V. For the capacitance, an Inductance-Capacitance-Resistance (LCR) digital bridge tester (Victor VC4090A) is utilized and the average value of the 8 mm length LN phase shifter is measured to be 39.5 pF. Consequently, the power consumption is calculated to be 1.11 nJ/π by Equation S3.
